# Alkaline Phosphomonoesterase-Harboring Microorganisms Mediate Soil Phosphorus Transformation With Stand Age in Chinese *Pinus massoniana* Plantations

**DOI:** 10.3389/fmicb.2020.571209

**Published:** 2020-11-27

**Authors:** Yueming Liang, Mingjin Li, Fujing Pan, Jiangming Ma, Zhangqi Yang, Tianwang Ling, Jiashuang Qin, Shaohao Lu, Fengyue Zhong, Zunrong Song

**Affiliations:** ^1^College of Environmental and Engineering, Guangxi Key Laboratory of Theory and Technology for Environmental Pollution Control, Guilin University of Technology, Guilin, China; ^2^Key Laboratory of Karst Dynamics, Ministry of Natural and Resources & Guangxi Zhuangzu Autonomy Region, Institute of Karst Geology, Chinese Academy of Geological Sciences, Guilin, China; ^3^Key Laboratory of Ecology of Rare and Endangered Species and Environmental Protection, Ministry of Education, Guangxi Normal University, Guilin, China; ^4^Production and Operation Department, Zhenlong Forest Farm of Hengxian County, Nanning, China; ^5^Engineering Research Center of Masson Pine of Guangxi, Guangxi Forestry Research Institute, Nanning, China; ^6^Guangxi Institute of Botany, Chinese Academy of Sciences, Guilin, China

**Keywords:** alkaline phosphomonoesterase, phoD-harboring microorganisms, P fractions, Pinus massoniana plantations, P-use strategies

## Abstract

*phoD*-harboring microorganisms facilitate mineralization of organic phosphorus (P), while their role in the regulation of soil P turnover under P-limited conditions in *Pinus massoniana* plantations is poorly understood. The aim of the present study was to investigate the effects of stand age and season on soil P fractions and *phoD*-harboring microorganism communities in a chronosequence of Chinese *P. massoniana* plantations including 3, 19, and 58 years. The soil P fractions (i.e., CaCl_2_-P, citrate-P, enzyme-P, and HCl-P) varied seasonally, with the higher values observed in the rainy season. The concentrations of the fractions were higher in old plantation (OP) soils and lower in young planation (YP) soils in both seasons. The OTU abundances were negatively correlated with total available P concentration, while were positively correlated with alkaline phosphomonoesterase (ALP) activity at 0–10 cm soil depth. The results indicate that *phoD*-harboring microorganisms have great potential to mineralize organic P under P-poor conditions and highlights those microorganisms are indicators of P bioavailability in *P. massoniana* plantations.

## Introduction

*Pinus massoniana* is a fast-growing native tree species in China and one of the important timber forest species in the region. The total area under *P. massoniana* plantations in China is approximately 1.001 × 10^8^ m^2^, and their stock volume is 5.91 × 10^9^ m^3^ (State Forestry Administration of China, 2013). *Pinus massoniana* plantations, similar to other forests, are often P-limited (Pan et al., 2020). Low soil P availability limits tree productivity, particularly in the tropics and subtropics, where highly weathered soils are widespread (Kochian, 2012). When trees are subjected to P-limitation in ecosystems, they adopt strategies to increase P supply, including increasing root exudate concentrations and promoting microbial activity (Wallander and Nylund, 1992; Wu et al., 2019b). Soil P availability may change with an increase in plant growth and stand age (Walker and del Moral, 2003; Bol et al., 2016; Liu et al., 2018). The age-related increase in stand productivity enhances trees P uptake (Hacker et al., 2015) and further reduces soil P bioavailability. In turn, as stand development, more litter input and more P returns into soil (Osman, 2013). Therefore, understanding the dynamics and mechanisms of soil P bioavailability with stand age could offer insights to forest managers on how to increase soil P availability and maintain productivity over the long-term in *P. massoniana* plantations. However, little attention has been paid to the underlying mechanisms influencing P bioavailability in *P. massoniana* plantations in stands of different ages.

P exists in soils in both inorganic and organic forms. Inorganic P (Pi) is usually composed of primary mineral-P (e.g., apatite), secondary crystalline and amorphous precipitates of Al/Fe, and P absorbed onto clay minerals (e.g., silicate) (Rodrigues et al., 2016). Organic P (Po) comprises orthophosphate monoesters (including inositol phosphates and orthophosphate diesters), organic polyphosphates, and phosphonates (Cui et al., 2015). The concentrations of the two P forms vary across soils hosting tree stands of different ages. For example, Pi in soils increased with stand development in Chinese Fir Plantations (Wu et al., 2019a). Po accounts for 30–70% of the total P in forest soils (Turner et al., 2007, 2013; Vitousek et al., 2010; Wu et al., 2019a), and it has to be mineralized to be available to plants through biological processes that are regulated by plants and soil microbes. Deluca et al. (2015) proposed a novel biological-based approach for evaluating P availability in complex ecosystems based on four P fractions (CaCl_2_-P, Citrate-P, HCl-P, and enzyme-P). This approach adopts four parallel extractions (CaCl_2_, HCl, a phytase and phosphatase mixture, and citric acid) to measure contents of four P fractions, which are defined as bioavailable P. Therefore, a better understanding of the P distribution within such fractions would facilitate the evaluation of P availability.

Soil microbes are key drivers of soil Po transformation processes owing to their capacity to synthesize phosphomonoesterase. Such synthesis processes are induced by P limitation, which stimulate microbes to upregulate the expression of phosphomonoesterase-encoding genes (Vershinina and Znamenskaya, 2002). The expression of such genes could reflect phosphomonoesterase activity in response to low Pi. Both alkaline phosphomonoesterase (ALP) and acid phosphomonoesterase could hydrolyze up to 89% of the total Po in soils (Kathuria and Martiny, 2011; Nannipieri et al., 2011; Jarosch et al., 2015). ALP is produced mainly by soil microbes (Nannipieri et al., 2011; Tan et al., 2013), and is, therefore, considered a key driver of bacterial P transformation. Although three homologous genes (*phoA*, *phoD*, and *phoX*) (Gomez and Ingram, 1995) regulate the synthesis of ALP, *phoD* is a key molecular marker applied in investigations on microbial P transformation processes owing to its ubiquitous distribution and greater abundance in diverse ecosystems (Tan et al., 2013; Ragot et al., 2015; Wei et al., 2019), as well as in acidic soils (Long et al., 2018; Wei et al., 2019) and alkaline soils (Hu et al., 2018).

Soil P availability in forest ecosystems could influence plant-*phoD*-harboring microorganism-soil interactions based on stand age. In forest ecosystems, P supply is maintained by the weathering of primary minerals and recycling of litter through decomposition. To maintain such equilibria, plants and microorganisms employ different P-use strategies. Lang et al. (2017) hypothesized that plants and microorganisms use P-recycling strategies at P-poor sites, while employing P acquiring strategies at P-rich sites. According to Lang’s hypothesis, plants and microorganisms growth are limited at P-poor sites. They would increase ALP activities mineralizing Po to Pi to sustain their P demand and synchronously minimize P losses from soils. In contrast, ALP activity would be inhibited (Wei et al., 2019), and plants and microorganisms transfer Pi into Po and immobilize P in P-rich soils. The P recycling and P acquisition strategies by plants and microorganisms suggests the change in P nutrition strategies depending on soil P availability. Therefore, soil P availability increases with an increase in stand age (Sharma et al., 2009; Gao et al., 2019; Wu et al., 2019a), which may lead to different dynamics in *phoD*-harboring microorganisms and ALP activity across different stand ages.

Seasonality influences the interaction between *phoD*-harboring microorganisms and soil P availability, owing to high variations in temperature, precipitation, and soil moisture. Previous studies have reported various seasonal dynamics of specific P fractions in different ecosystems (Picone et al., 2003; Yang et al., 2010; Liu et al., 2018). McGrath et al. (2000) reported that soil available P concentrations were the highest in the early rainy season in a peach palm-cupuassu agroforest in the Amazon. Conversely, soil available P concentrations were the lowest in the rainy season in tropical rain forests of Costa Rica (Cleveland et al., 2004). Such discrepancies could be explained by seasonal fluctuations in *phoD*-harboring microbial activities that influence soil P availability and ecosystem types. Therefore, the investigation of seasonal variations in soil P fractions could facilitate our understanding of soil P availability dynamics, in addition to how soil microbes and P acquisition strategies of trees regulate Po mineralization-immobilization processes (Chen et al., 2003). Considering most of the previous studies on the seasonal dynamics of soil P have been conducted in humid or semi-arid temperate regions (Styles and Coxon, 2007; Zhao et al., 2009), relatively little information is available on the seasonal dynamics of soil P fractions and *phoD*-harboring microorganisms in subtropical regions, which are characterized by marked dry and wet seasons.

Soil profile is a key factor influencing the bacterial community structure directly by changes of resources and indirectly changes of habitats (Senbayram et al., 2018; Yu et al., 2019). Soil profile represents a strong ecological filter for selecting soil microorganisms. Agnelli et al. (2004) have been reported that some microbial taxa capable of utilizing soil organic carbon are prevalence in deep soil layer due to higher soil microbial biomass carbon and organic carbon ratio. The higher ratio indicates higher use efficiency of organic carbon by microorganism or the higher portion of carbon from shoots. The partitioning of soil organic P along soil profiles is also observed in different stand ages of rubber-based agroforestry (Liu et al., 2018), however, information about soil *phoD*-harboring microorganisms transformation organic P to inorganic P as responses to low P environment for plant growth is little. Therefore, more attentions should be paid to understand the partitioning mechanisms of soil organic P by *phoD*-harboring microorganisms along soil profiles.

As demand for timber increased rapidly in the 1960s, large areas of Chinese *P. massoniana* plantations were established in the south of China, including our present sites (one of the major *P. massoniana* production areas). A chronosequence (space-for-time substitution) approach was also applied in the selection of sites in the present study, which had been adopted in numerous previous studies (Walker et al., 2010; Wu et al., 2019a). In this study, our aim was to explore how stand development influences soil P fractions and *phoD*-harboring microorganisms in both rainy and dry seasons in a chronosequence of *P. massoniana* plantations. Soil P fractions, ALP activity, and *phoD*-harboring microbial communities were examined. We hypothesized that (1) the *phoD*-harboring microbial communities and ALP activity variables were related to the changes in soil P availability with an increase in stand ages and (2) soil P supply influences P nutrition strategies of *P. massoniana* plantations in Southwestern China.

## Materials and Methods

### Site Description and Experimental Design

**Figure 1:**
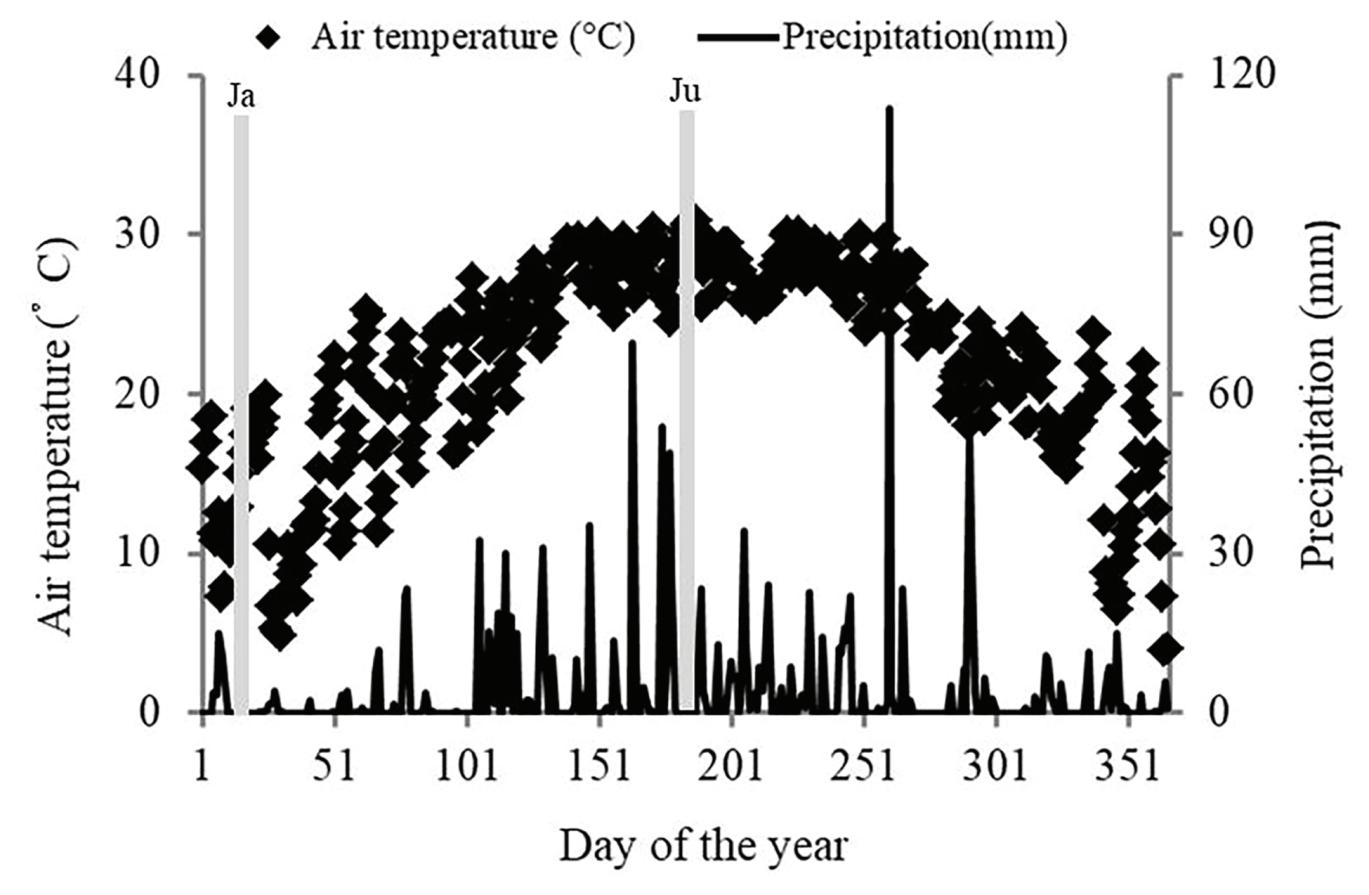
Meteorological conditions at the study site in 2018. The two gray areas represent the two sampling periods, Ja and Ju, (January and July, respectively), which represent the dry and rainy seasons, respectively. The data were provided by the weather bureau of Hengxian.

The study was conducted in Zhenlong State Tree Farm (22°08'–23°30'N and 108°48'–109°37'E), Guangxi Zhuang Autonomous Region, Southwestern China. The region has a subtropical monsoon climate. The mean annual temperature is 22°C and the mean annual precipitation is 1,450 mm. During the study period (January 1 to December 31, 2018), the site received 1,352 mm of rainfall (Figure 1). The dry season began in the beginning of January 2018 and continued until mid-March 2018. The rainy season occurs from April to September 2018, although there were a few days of heavy rain in October. The maximum monthly temperature was 28°C (July) and the minimum monthly temperature was 13°C (January). Soil type is classified as laterite according to the FAO-UNESCO Soil Classification System.

As demand for timber increased rapidly in the 1960s, the Chinese government has implemented policies for increasing timber production. Cropland in our sites has been abandoned and large areas of Chinese *P. massoniana* plantations were established in the south of China, including our present sites (one of the major *P. massoniana* production areas). Thus, a chronosequence of Chinese *P. massoniana* plantation characterized by different abandoned ages has selected. In brief, three stand ages of Chinese *P. massoniana* plantation were selected and represented young plantations (YP; 6-year-old), middle-aged plantations (MP; 19-year-old), and over-mature plantations (OP; 58-year-old). Three sites (20 m × 20 m) for each stand, at least 100 m apart from each other, were established. All sites were located near mid-slope positions, and there were minor differences among the selected sites in gradient (16–29°), aspect, and altitude (315–377 m). In total, nine sites (three stand ages × three replicate sites) were established. The selected sites had experienced minimal impact after plantation because Zhenlong State Tree Farm established in 1957. Within the first 3 years of planting, no management measures were undertaken except for the manual removal of herbs and shrubs to facilitate seedling growth. The dominant understory shrubs in *P. massoniana* plantations included *Melastoma normale*, *Wendlandia aberrans*, *Evodia lepta*, *Schefflera heptaphylla*, and *Ardisia quinquegona*, while dominant herbs include *Miscanthus floridulus*, *Rubus alceifolius*, and *Smilax china*. Descriptions of stand characteristics are presented in Supplementary Table S1.

### Field Sampling

Sampling was conducted in January (dry season) and July (rainy season) 2018. In each site (20 m × 20 m), three 50 cm × 50 cm areas were randomly established, and floor litter was collected in both seasons. Three floor litter samples from a site were combined to form a composite sample, and then oven-dried at 60°C for subsequent analysis of litter P (Supplementary Figure S1). After the litter was collected, soil cores were obtained from each site at 0–10, 10–20, and 20–30-cm depths using a 5-cm-diameter auger in three stands. The sampling depth was determined according to the spatial distribution of tree roots in this region (Song et al., 2020). Five soil cores based on an “S” shape were obtained from the same depth in a site and combined to form a composite sample in the field and immediately transported to the laboratory. Soil samples were sieved through 2-mm sieves to remove stones, animals, roots, and plant material. One subsample (10 g) was immediately stored at −80°C for use in subsequent *phoD*-harboring microbial community analyses. One subsample was maintained at 4°C for use in enzyme activity, microbial biomass P (MBP), and soil P fraction analyses. After collection, the CaCl_2_-P, citrate-P, HCl-P, and MBP were measured within 4 weeks, and enzyme-P and enzyme activity were determined within 2 weeks. The remaining subsamples were air-dried for use in soil physicochemical property analyses.

### General Soil Parameters

Soil pH was determined using suspensions of the samples in water at a ratio of 1:2.5 (w/v) using a Mettler Toledo 320 pH meter (Delta 320; Mettler-Toledo Instruments Ltd., Shanghai, China). Total nitrogen (TN) was measured using a FIAstar (FIAstar 5,000 FOSS, Sweden Ltd) based on the Kjeldahl method (Bremner, 1956). Soil organic carbon (SOC) was measured using the K_2_Cr_2_O_7_-H_2_SO_4_ oxidation-reduction titration method. Total P (TP) was determined by acid digestion using a H_2_SO_4_ + HClO_4_ solution. Exchangeable magnesium (Mg) and calcium (Ca) were displaced *via* compulsive exchange in 1 mol L^−1^ ammonium acetate at pH 7.0 and analyzed using inductively coupled plasma atomic emission spectroscopy (ICP-AES; Carter and Gregorich, 2006). The soil physicochemical properties above are listed in Supplementary Table S2. Olsen-P was extracted with 0.5 M NaHCO_3_ and measured using the ammonium molybdate method (Olsen et al., 1954), while MBP was determined using the CHCl_3_ fumigation extraction method (Wu et al., 1990).

ALP activity in soils was measured by fluorometric method according to Finzi et al. (2006). The methylumbelliferyl phosphate (MUP, Sigma) was as a fluorogenic substrate. Soil suspensions were prepared by homogenizing 1 g fresh soil using 125 ml 50 mM sodium bicarbonate buffer (pH 9.0). Briefly, the 96 wells were divided into sample assay (200 μl soil suspensions + 50 μl 200 μM MUP-linked substrate), soil control (50 μl buffer + 200 μl soil suspensions), quench standard (200 μl soil suspensions + 50 μl 10 μmol L^−1^ MUP), reference standard (200 μl buffer + 50 μl 10 μM MUP), negative control (200 μl buffer + 50 μl 200 μM MUP-linked substrate), and blank wells (250 μl buffer). The microplates were incubated in the dark at 25°C for 4 h and 10 μl 1.0 M NaOH was added to each well to halt reactions. Fluorescence was determined using a microplate fluorometer (Infinite 200 Pro, Tecan, Switzerland) at 365 and 450 nm excitation and emission wavelengths, respectively. ALP activity was calculated as nmol 4-methylumbelliferone (MUF) g^−1^ soil h^−1^.

### Phosphorus Fractionation

Four P fractions, including CaCl_2_-P, citrate-P, enzyme-P, and HCl-P, were measured using the biologically based P extraction method according to DeLuca et al. (2015). CaCl_2_ extractable P represents Pi that is easily available to plants, while enzyme extractable P represents available Po that is hydrolyzed by phytase and phosphatase. Citrate extractable P represents potential soluble Pi, which would be accessible to plants when soil organic acids are released into the soil. In addition, HCl extractable P represents recalcitrant Pi, which can be solubilized by proton excretion released by plant and microbes. Each of the P fractions was measured by shaking 0.5 g of fresh soil with 10 ml of extractant (10 mM CaCl_2_ for CaCl_2_-P, 0.2 U enzymes for enzyme-P, 10 mM citric acid for citrate-P, and 1 M HCl for HCl-P) in separate 15-ml centrifuge tubes on a reciprocal shaker at 200 rpm for 3 h. Extracts were centrifuged at 3000 × *g* for 5 min, and then all of the supernatant was determined by the malachite green method at 630 nm (Ohno and Zibilske, 1991) using a PowerWave-XS microplate spectrophotometer (Infinite M200 PRO, Switzerland). The enzyme extractant consisted of three enzymes: 0.5 U acid phosphomonoesterase (Sigma P3627), 0.5 U alkaline phosphomonoesterase (Sigma P5931), and 0.1 U phytase (Sigma P5931).

### DNA Extraction and Illumina Sequencing

Soil DNA was extracted from 0.5 g of frozen soil using a FastDNA SPIN kit for soil (MP Biomedicals, Cleveland, OH, United States) according to the manufacturer’s instructions. The quantity and quality of extracted DNA were measured using a Nanodrop ND-1000 UV/vis spectrophotometer (NanoDrop Technologies, Wilmington, DE, United States) and then examined on a 1% (w/v) agarose gel. Primers ALPS-F730 and ALPS-R1101 (Sakurai et al., 2008) labeled with a unique barcode at the 5' end were used to amplify *phoD* and to distinguish the sequences of each sample. Amplification of each sample was performed in triplicate in a 25-μl reaction including 2.5 μl 10 × Ex Taq buffer (Mg^2+^ plus), 0.3 μl Ex Taq (Takara, Japan), 2 μl DNA, 0.5 μl of each primer, and 19.2 μl ddH_2_O. The PCR was performed under the following cycling conditions: 95°C for 3 min, followed by 30 cycles at 95°C for 30 s, 57°C for 30 s, and 72°C for 30 s, and a final extension at 72°C for 10 min. The PCR products were then purified using the TIANquick Midi Purification Kit (TIANGEN, China). Sequencing was performed on an Illumina HiSeq2500 platform by Magigene Co., Ltd. (Guangzhou, China).

### Analysis of Illumina Sequencing Data

The sequences were processed using the QIIME platform (Caporaso et al., 2010). Raw sequences were quality screened, and sequences shorter than 200 bp, with average quality scores lower than 30, and containing any ambiguous bases were discarded. Subsequently, sequences with chimeras were removed using UCHIME v9.0 methods in the QIIME 1 platform. The remaining sequences of nucleotides converted to amino acid sequences that did not match *phoD* or had a termination codon were removed using the FrameBot tool in the RDP function gene pipeline.^1^ The obtained high-quality sequences were clustered into operational taxonomic units (OTU) using UCluster at 75% similarity (Tan et al., 2013; Wei et al., 2019). Subsequently, taxonomy assignment of each OTU was performed using BLAST in the Fun-Gene database (Fish et al., 2013). The starting compositional analyses are log-ratio (clr) transformation abundance of OTU data (Aitchison, 1983; Gloor et al., 2017). Estimating the 0 count values were using zCompositions R package (Palarea-Albaladejo and Martín-Fernández, 2015). Alpha-diversity and *β*-diversity was calculated according to log-ratio (clr) data. For dominant OTUs (each dominant OTU accounting for 8% of the total sequences), a representative sequence was queried against the GenBank database using BLAST, which was, in turn, used to construct a maximum likelihood phylogenetic tree using MEGA 7 (Supplementary Figure S2). The sequence data have been deposited in the NCBI database under BioProject accession number SRR11318226.

### Plant Litter P and Soil P Pool Estimation

Litter P pool was calculated using the following equation: TPS litter (kg ha^−1^) = WA × TPcon, where TPS litter is the P pool of the litter (t ha^−1^), WA is the weight of litter per unit area (g m^−2^), and TPcon is the litter P concentration (g kg^−1^). Annual tree P requirements were estimated according to the methods of Johnson et al. (2003), who estimated that litter P accounts for c. 60% of annual tree P requirements on based on data from temperate forests. Soil P pools were calculated using the following equation: TPSsoil (kg ha^−1^) = BD × TPcon, where B is the soil bulk density (g cm^−3^) and D is the soil depth (cm).

### Statistical Analysis

Data (i.e., P concentrations and physico-chemical properties) were tested for normality using IBM SPSS Statistics 17 (IBM Corp., Armonk, NY, United States). If data were not satisfied normality and homoscedasticity tests, and log-transformed were performed. Differences in P concentrations and physico-chemical properties in each stand age for the same season were tested using one-way ANOVA and Duncan’s multiple range *post hoc* tests at *p* < 0.05. Two-way ANOVA was used to test the effects of stand age, sampling season, and their interactions on soil P fractions. The non-parametric multivariate statistical test of dissimilarity (MRPP) was used to evaluate variations in the composition of *phoD*-harboring microbial communities among treatments using the vegan package in R version 3.5.1. Furthermore, principal component analysis (PCA) was performed to detect dissimilarity in *phoD*-harboring microorganisms using the vegan package in R version 3.5.1. Redundancy analysis (RDA) was performed to identify the major factors influencing the composition of *phoD*-harboring microbial communities using CANOCO (version 5.0, Microcomputer Power, Inc., Ithaca, NY, United States). Environmental factors, including SOC, pH, TN, TP, MBP, ALP activity, CaCl_2_-P, citrate-P, enzyme-P, HCl-P, and exchangeable Mg and Ca, were used in RDA.

## Results

### Soil P Fractions and ALP Activity

**Figure 2:**
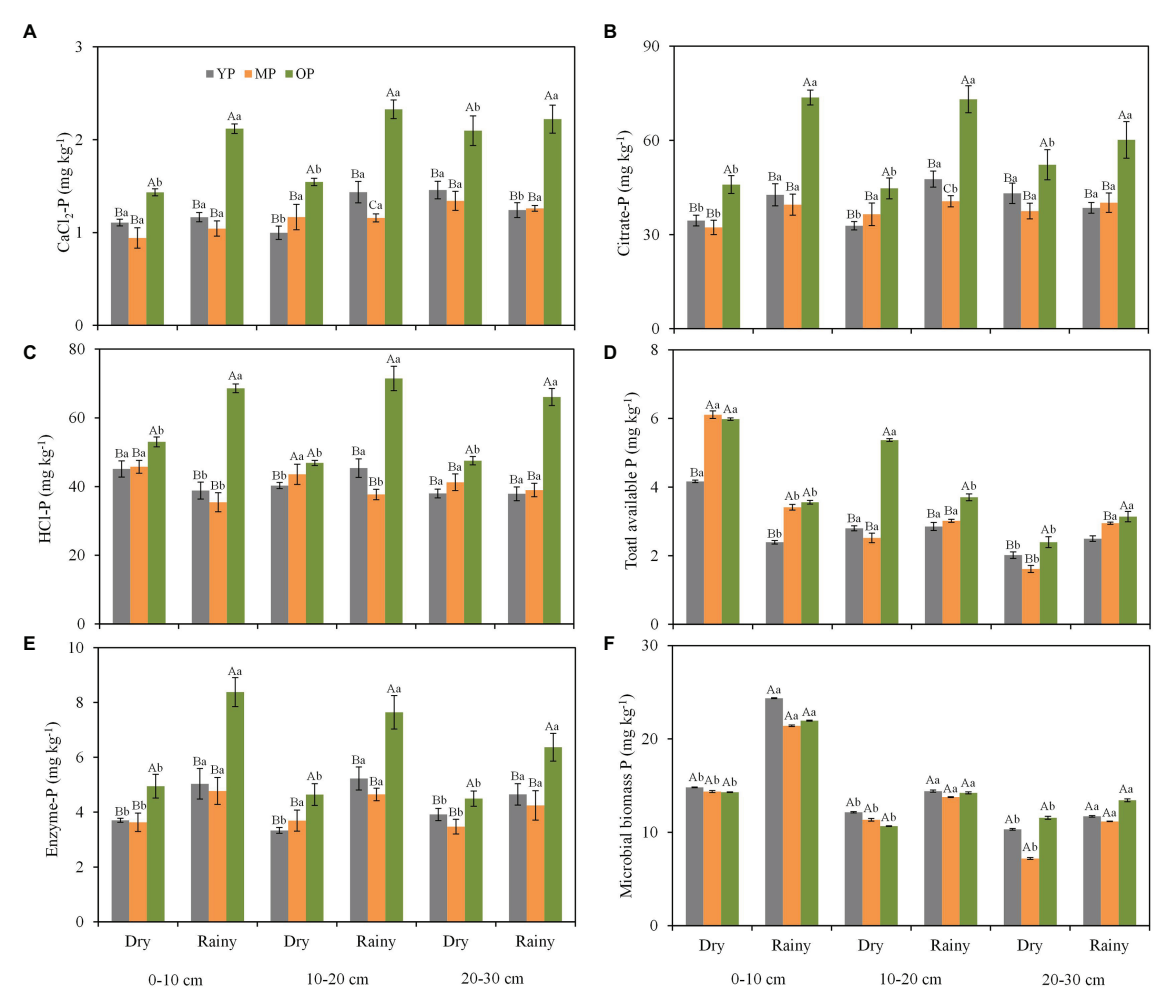
Seasonal changes in CaCl_2_-P **(A)**, citrate-P **(B)**, HCl-P **(C)**, total available P **(D)**, enzyme-P **(E)**, and microbial biomass P **(F)** fractions at different soil layers and stand ages in *P. massoniana* plantations. Error bars indicate standard errors of the mean (*n* = 3). Values within each sampling season followed by different lowercase letters differ significantly according to Duncan’s multiple range test (*p* < 0.05). Values within each stand age followed by different capital letters differ significantly according to Duncan’s multiple range tests (*p* < 0.05). YP, young plantations; MP, middle-aged plantations; and OP, over-mature plantations. Different capital case letters are significantly different among stand ages according to Duncan test (*p* < 0.05). Different lower case letters are significantly different between seasons according to Duncan test (*p* < 0.05).

Stand age and season influenced soil P fraction concentrations while soil depth had minimal effect on soil P fractions. The concentrations of all the soil P fractions (i.e., CaCl_2_-P, citrate-P, enzyme-P, HCl-P, and soil total available P) were higher in the over-mature plantations than in the young and middle-aged plantations (Figures 2A–F), except for MBP concentrations (Figure 2F). In addition, soil total available P in the dry season was 2-fold the concentration in the rainy season at 0–10 cm soil layer (Figure 2D). The concentrations of enzyme extractable P and MBP were lower in the dry season than in the rainy season at three soil layers (Figures 2E,F). Furthermore, significant interaction effects of stand age and season on soil P pools (i.e., CaCl_2_-P, citrate-P, enzyme-P, and HCl-P) were observed in the 0–10 cm soil layer (Supplementary Table S3). Simultaneously, significant interaction effects of stand age and season on citrate extractable P concentration were observed in the 20–30 cm soil layer (Supplementary Table S3).

**Figure 3:**
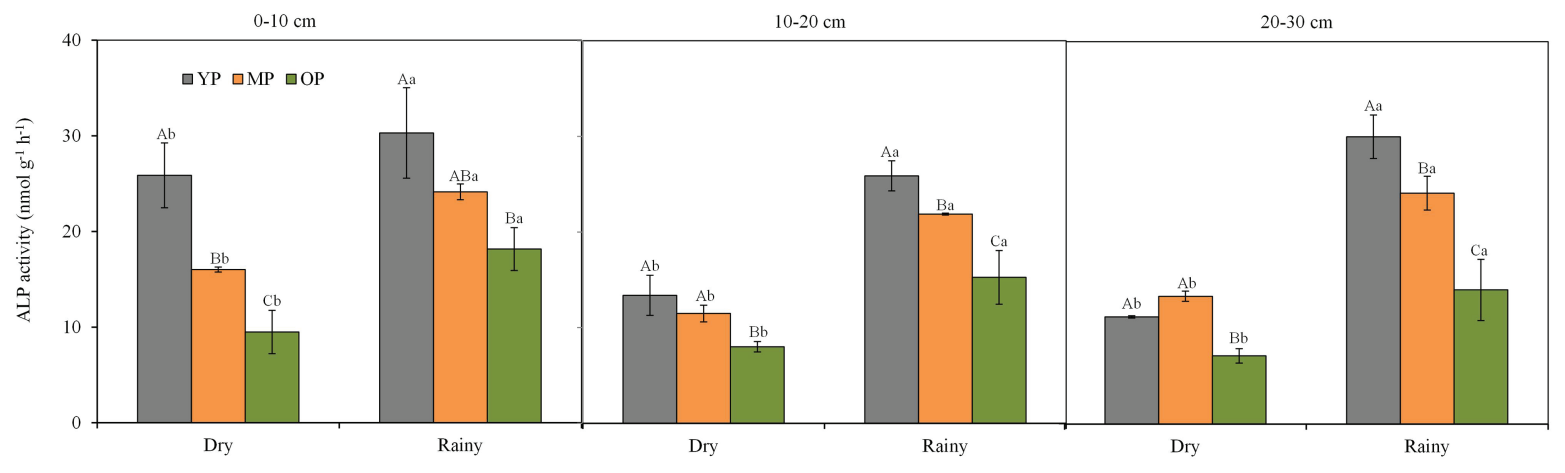
Seasonal changes in soil ALP activity at different soil layers and stand ages of *P. massoniana* plantations. Error bars indicate standard errors of the mean (*n* = 3). Values within each sampling season followed by different lowercase letters differ significantly according to Duncan test (*p* < 0.05). Values within each stand age followed by different capital letters differ significantly according to Duncan’s multiple range test (*p* < 0.05). ALP, alkaline phosphatase; YP, young plantations, MP, middle-aged plantations; and OP, over-mature plantations. Different capital case letters are significantly different among stand ages according to Duncan test (*p* < 0.05). Different lower case letters are significantly different between seasons according to Duncan test (*p* < 0.05).

Soil ALP activity was higher in the young plantation soils but lower in the over-mature plantations soils (Figure 3). In addition, soil ALP activity varied seasonally at three soil depths (*p* < 0.05), with higher values observed in the rainy season when compared with the dry season. Significant negative correlations were observed between total available P concentration and ALP activity at 0–10 and 10–20 cm soil depths.

### Effects of Stand Ages on P Pools

**Figure 4:**
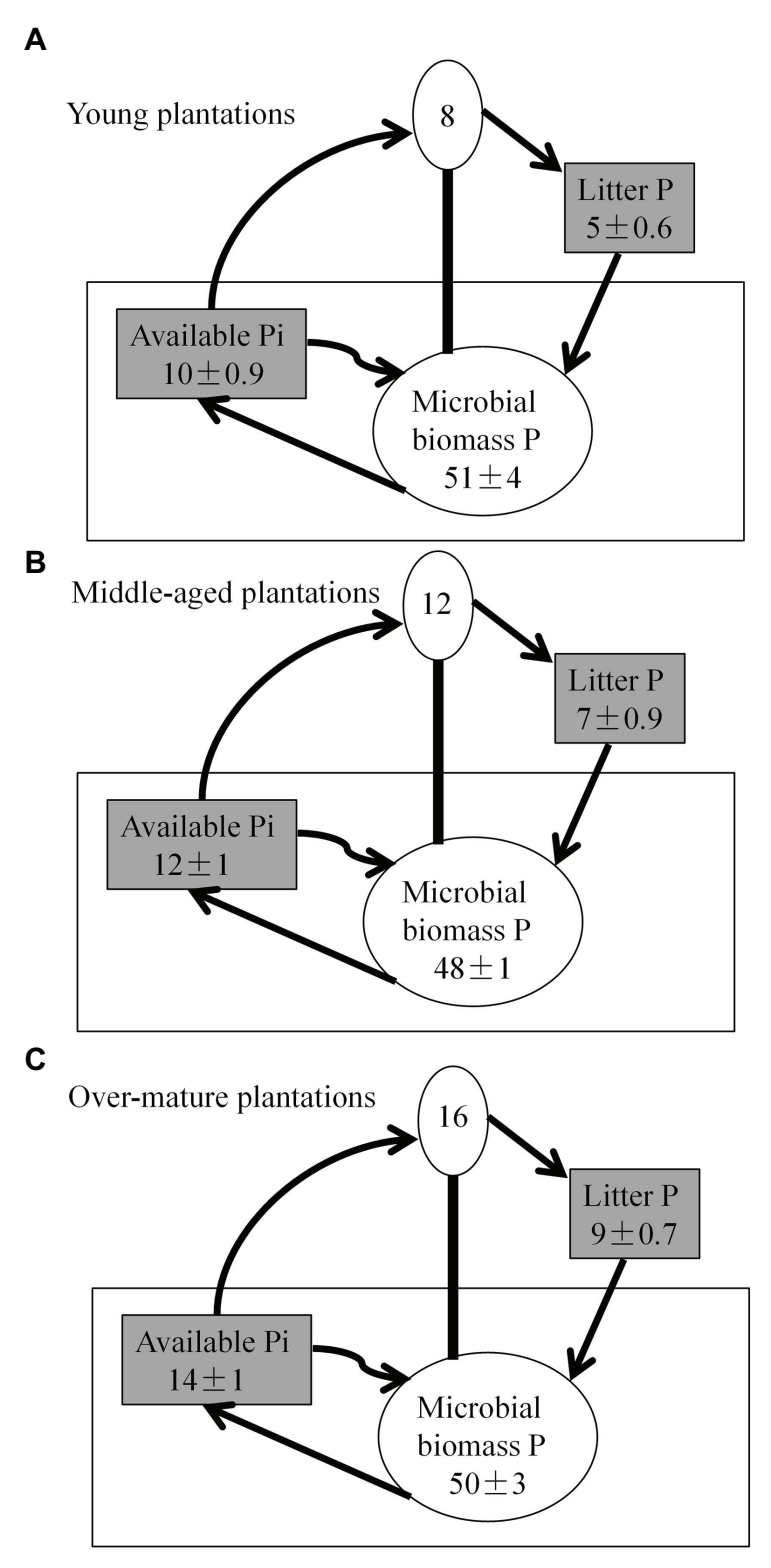
Average phosphorus (P) pools scaled to illustrate relative pools sizes in kg P ha-1 down to 30 cm soil depth. Litter and soil available inorganic P (Pi) pools are gray to illustrate that these were significantly different between **(A)** young plantations, **(B)** middle-aged plantations, and **(C)** over-mature plantations. Biotic pools (annual tree requirement and microbial biomass) are illustrated as circles. Annual tree P requirements are estimated according to the litter P. Available Pi represents bicarbonate extracted pools. Arrows illustrate the proposed main fluxes in the three stand types. Values, except for tree P requirements, are the seasonal averages ± SE.

Soil available P pool and litter P pool were higher in the over-mature plantations than in the young and middle-aged plantations (*p* < 0.05; Figure 4). However, soil MBP did not vary significantly among the three stands. Annual tree P requirements accounted for approximately one-fourth of the annual MBP. The sum of annual average MBP and estimated annual tree P requirements exceeded the soil available P pools.

### Diversity and Community Structure of *phoD*-Harboring Microorganisms

After discarding chimeras, non-target *phoD* sequences, and rarifying random sequences to 20,832 per sample, 307 OTUs were recovered based on a 75% similarity cluster threshold. The Shannon diversity index of *phoD*-harboring microorganisms were higher in the over-mature plantations and lower in middle-aged plantations (Supplementary Table S4).

**Figure 5:**
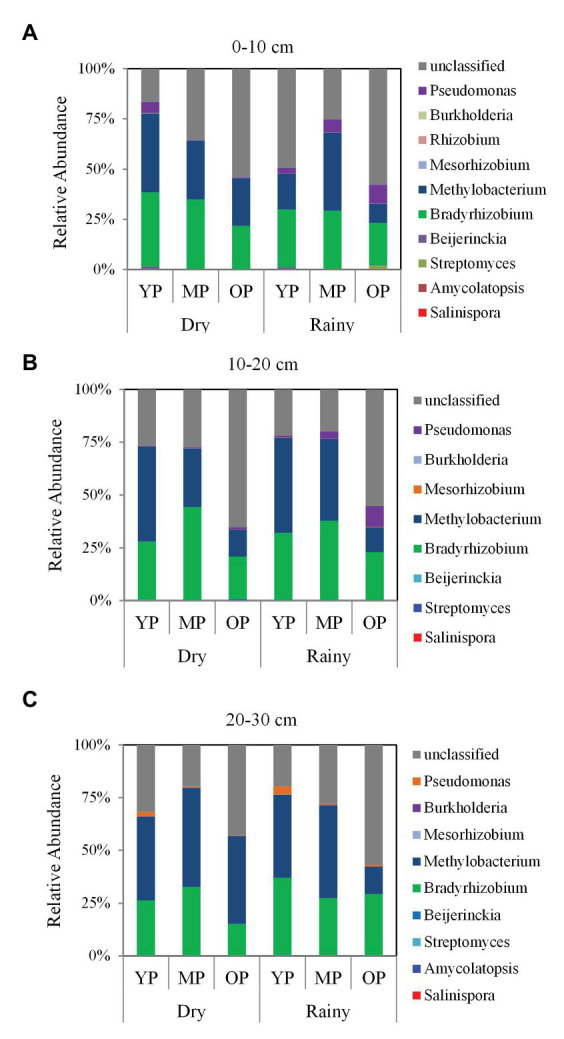
Taxonomic composition of *phoD*-harboring microbial communities at different soil layers **(A)** 0–10 cm, **(B)** 10–20 cm, and **(C)** 20–30 cm at the genus level in three *P. massoniana* plantations of different ages. YP, young plantations; MP, middle-aged plantations; and OP, over-mature plantations.

The highest relative abundances of phyla were Proteobacteria and Cyanobacteria, and the highest relative abundances of orders were Burkholderiales and Rhizobiales (Supplementary Figure S3). In addition, the highest relative abundances of genera (>8%) were *Bradyrhizobium* and *Methylibium* in the three stands (Figure 5). The relative abundance of *Bradyrhizobium* and *Methylibium* at the 0–10 and 10–20 cm soil layers were higher in the young and middle-aged plantations, while were lower in the over-mature plantations (Supplementary Figure S4). However, season had negligible effects on the relative abundances of the two dominant genera (Supplementary Figure S4).

### Factors Affecting *phoD*-Harboring Microbial Community Structure

OTU abundance was negatively correlated with total available P concentration (*R* = −0.495, *p* = 0.037), while was positively correlated with ALP activity (*R* = 0.528, *p* = 0.024) at 0–10 cm soil depth. Significant negatively correlations were observed between OTU abundance and Enzyme-P (R = −0.461, *p* = 0.047), CaCl_2_-P (*R* = −0.588, *p* = 0.008), Citrate-P (*R* = −0.506, *p* = 0.027), and HCl-P (*R* = −0.520, *p* = 0.022) at 10–20 cm soil depth, respectively.

Based on the results of MRPP analyses (Table 1), stand age and season influenced *phoD*-harboring microbial community structure. In addition, PCA analysis facilitated the visualization of differences in *phoD*-harboring microbial community structure among the three stands (Supplementary Figure S5). The *phoD*-harboring microbial community structures significantly differed between two seasons at three soil depths. The community structure in young plantation soils was similar to over-mature plantation soils, while differed from middle-aged plantation soils at 0–10 and 10–20 cm soil depths. The community structure among three stand ages became similar at 20–30 cm soil depth.

**Table 1:** Significance testing results based on non-parametric multivariate statistical approaches (MRPP) to assess the effects of treatments on *phoD*-harboring microbial community composition.

Comparisons		MRPP		
		Observed δ	Expected δ	*p*
0–10 cm	YP vs. MP	0.475	0.505	0.06
	YP vs. OP	0.49	0.541	**0.002**
	MP vs. OP	0.485	0.537	**0.022**
	Dry vs. Rainy	0.539	0.54	**0.030**
10–20 cm	YP vs. MP	0.46	0.522	**0.02**
	YP vs. OP	0.476	0.542	**0.013**
	MP vs. OP	0.642	0.617	0.826
	Dry vs. Rainy	0.532	0.528	**0.038**
20–30 cm	YP vs. MP	0.35	0.361	0.151
	YP vs. OP	0.447	0.519	**0.001**
	MP vs. OP	0.425	0.522	**0.003**
	Dry vs. Rainy	0.486	0.485	0.386

The difference is significant when at least two tests yield *p* < 0.05 (bold).

**Figure 6:**
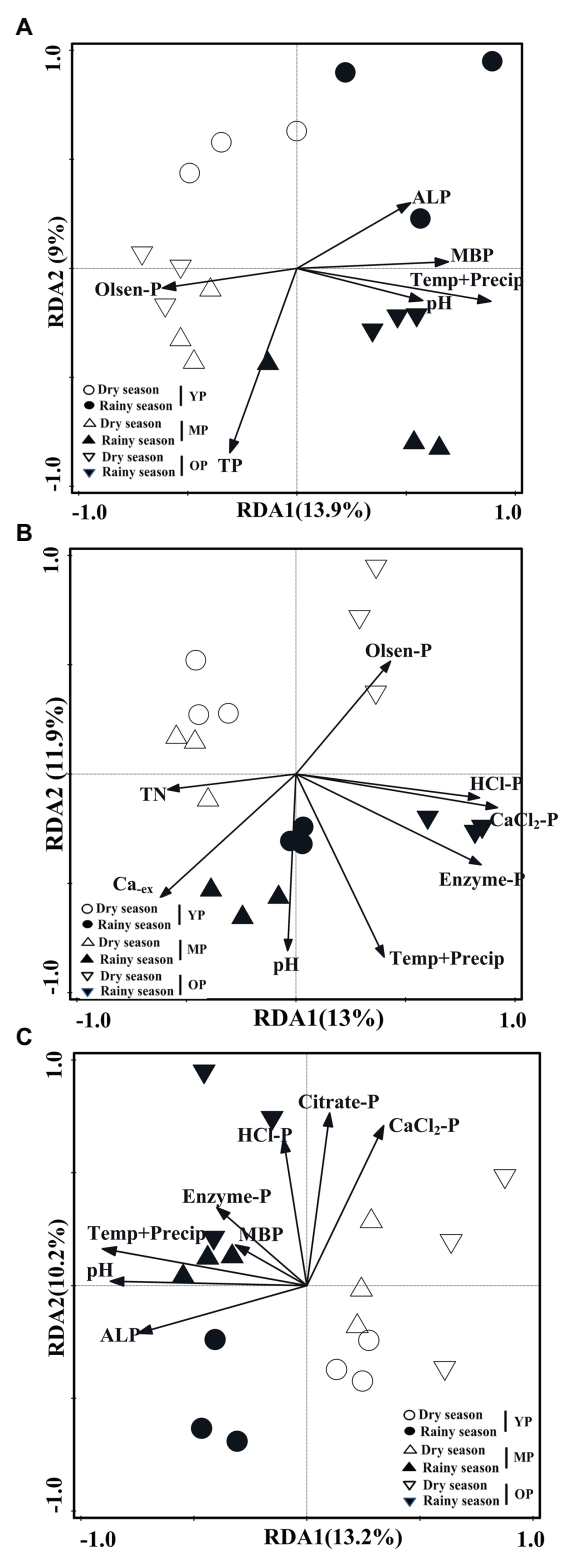
RDA showing the relationships between soil properties and the OTU profiles of *phoD* gene 0–10 cm **(A)**, 10–20 cm **(B)**, and 20–30 cm **(C)**. Displayed vectors represent the environment factors that are significantly correlated to the community structures. RDA, Redundancy analysis; OTU, Operational Taxonomic Units; TN, total nitrogen; Ca_ex_, exchangeable Ca; TP, total phosphorus; Olsen-P, total availability phosphorus; MBP, microbial biomass phosphorus; CaCl_2_-P, CaCl_2_ extractable phosphorus; citrate-P, citrate extractable phosphorus; enzyme-P, enzyme extractable phosphorus; HCl-P, HCl extractable phosphorus; ALP, alkaline phosphatase; Temp+Precip, air temperature and Precipitation; YP, young plantations; MP, middle-aged plantations; and OP, over-mature plantations.

RDA analysis revealed that *phoD*-harboring microbial community structure at 0–10 cm soil depth was significantly affected by the interaction effects of air temperature and precipitation (*F* = 2.2, *p* = 0.001), MBP (*F* = 1.62, *p* = 0.01), TP (*F* = 1.5, *p* = 0.032), pH (F = 1.5, *p* = 0.026), ALP (F = 1.5, *p* = 0.037), and Olsen-P (F = 1.5, *p* = 0.041), while they accounted for 22.90% of the total variance in the *phoD* OTUs profile (Figure 6A). The *phoD*-harboring microbial community structure at 10–20 cm soil depth was significantly affected by CaCl_2_-P (*F* = 1.9, *p* = 0.001), Enzyme-P (*F* = 1.8, *p* = 0.002), interaction effects of air temperature and precipitation (*F* = 1.7, *p* = 0.003), HCl-P (F = 1.7, *p* = 0.004), Ca_ex_ (F = 1.6, *p* = 0.001), pH (F = 1.5, *p* = 0.019), TN (*F* = 1.4, *p* = 0.019), and Olsen-P (F = 1.4, *p* = 0.043), and they accounted for 24.90% of the total variance in the *phoD* OTUs profile (Figure 6B). The *phoD*-harboring microbial community structure at 10–20 cm soil depth was significantly affected by the interaction effects of air temperature and precipitation (F = 2.2, *p* = 0.001), pH (*F* = 2.1, *p* = 0.001), ALP (F = 1.8, *p* = 0.003), CaCl_2_-P (F = 1.7, *p* = 0.002), Citrate-P (F = 1.5, *p* = 0.019), MBP (F = 1.5, *p* = 0.011), Enzyme-P (F = 1.4, *p* = 0.032), and HCl-P (F = 1.4, *p* = 0.04), and these variables explained 23.4% of the total variance in this *phoD* OTU profile (Figure 6C).

## Discussion

### Shifts in Soil P Fractions in *Pinus massoniana* Plantations With Stand Age

Understanding the seasonal dynamics of bioavailable P under different stand ages along the soil profile could facilitate sustainable forest management; however, few studies have examined such dynamics. Soil P fractions (i.e., total available P, CaCl_2_-P, citrate-P, enzyme-P, and HCl-P) differed markedly across the three stands with different ages, and their concentrations were higher in the over-mature plantations and lower in the young plantations. Differences in litter quantity and quality drive changes in soil P availability in forest ecosystems (Becker et al., 2015; Lang et al., 2016; Wu et al., 2019a). At the ecosystem scale, understory vegetation is an important input source of litter (Grierson and Adams, 2000). A previous study about vegetation investigation in our present sites shows that understory shrubs are more abundant in old and mature plantations than in young plantations (Qin, 2019). This result suggested that more litter from the understory vegetation return to the soil in old and mature plantations, accompanying with higher litter P content in old-mature plantations. Higher P nutrient inputs from litter decomposition increased soil P availability in old-mature planation, which is consistent with the findings of a previous study in Chinese fir forest soils (Wu et al., 2019a).

Soil P availability is influenced by concentration of pH and Ca^2+^ through precipitation and dissolution dynamics (Schafer, 1963; Traina et al., 1987; Hosseinpur et al., 2012). An increasing in soil P availability was observed when a decreasing in soil pH and exchangeable Ca^2+^ concentration from young plantations to over-mature plantations in our present study. The result can be explained by two main reasons. Firstly, protons (i.e., H^+^) concentration increases with a decreasing in soil pH, which contributes to Pi dissolution (Joos and Black, 1950). Secondly, exchangeable Ca^2+^ conduces to calcium phosphate precipitation (Tunesi et al., 1999). Thus, a decreasing in Ca^2+^ concentration in over-mature plantations would induce the Pi dissolution from a Ca/Fe-phosphate mineral.

Previous studies have demonstrated that strong rain-drought seasonality may play a key role in seasonal P cycling dynamics in ecosystems through the release of P in rainy season and the immobilization of P in dry season (Lopez-Gutierrez et al., 2004; Turner et al., 2015; Liu et al., 2018). We also observed higher available P in dry season and lower in rainy season at 0–10 cm depth in three stand ages. This result was partly further supported by seasonal changes of litter P content. In addition, citrate extractable P and enzyme extractable P pools varied significantly across seasons, suggesting that the pools play important roles in seasonal Pi cycling. We observed more notable seasonal dynamics of the four P pools (i.e., CaCl_2_-P, citrate-P, enzyme-P, and HCl-P) studied in the over-mature planation soils across the soil profile, suggesting that soil P cycling was more active in over-mature planation.

### Shifts in *phoD*-Harboring Microbial Community Structure With Stand Age

According to the results of our study, the dominant genera in different stands were *Bradyrhizobium* and *Methylibium*. *Bradyrhizobium*, a free-living and symbiotic dinitrogen (N_2_)-fixer (Kaneko et al., 2002), was dominant across three stand ages, suggesting that the genus couples soil P and N cycling processes. Notably, some *Bradyrhizobium* species respond sensitively to P and N limitation (Sakurai et al., 2008; Wei et al., 2019). Such coupling could enhance growth and development in *P. massoniana* ecosystems, where trees may experience P and N stress. In additionally, rare taxa (i.e., *Rhizobium* and *Pseudomonas*) have also been reported to play key roles in soil P and N cycling processes (Ishaq et al., 2020). The previous studies have been reported that *Methylibium* prefer conditions in which P concentrations insufficient (Ahn et al., 2006; Veraart et al., 2015). Simultaneously, this genus can exploit methane as the sole C source during growth, in turn, decreasing methane emissions from the soil (Hristova et al., 2003; Kane et al., 2007). Therefore, the dominant genera in the present study not only facilitate ALP hydrolysis of Po into Pi under P-limited conditions but also contribute to C and N cycling, suggesting that future research should be considered the coupling processes between P and C/N cycling.

Bacterial communities exhibit shifting trends associated with soil properties across different stand ages (van Der Heijden et al., 2008; Williams et al., 2013). In the present study, *phoD*-harboring microbial community structures were influenced by various soil P fractions in three stand ages. The results indicated a potential role of the *phoD*-harboring microorganisms in the mineralization of Po in P-poor conditions within *P. massoniana* plantations. For example, higher relative abundances of *Bradyrhizobium* and *Methylibium* are accompanied with lower P concentrations, according to many previous studies (Sakurai et al., 2008; Veraart et al., 2015; Wei et al., 2019). In addition, P limitation simulates *phoD*-harboring microorganisms to synthesize and secrete ALP (Santos-Beneit, 2015; Bergkemper et al., 2016; Ge et al., 2017), which mineralizes Po into Pi and, in turn, increases soil P availability.

A negative correlation between OTU abundance and total available P at 0–10 cm soil depth was found in present study. The total available P content was higher in old-mature plantation soils but lower in young plantation soils, probably because lots of litter input and less of P consumption by microorganisms in over-old plantation. Simultaneously, this increase in total available P content was accompanied by a significant decrease in ALP activity. This was because that ALP activity is sensitive to P availability, and the synthesis of ALP was repressed by higher available P content (Apel et al., 2007). The result suggested that microbial production of phosphatases is inhibited by inorganic P content (Nannipieri et al., 2011; Zhu et al., 2018). Additionally, the OTU abundance exhibited the most significant negative correlation with HCl extractable P at 10–20 soil depth, which represents the hardly available inorganic P. The results indicated that many inorganic P might be fixed in a recalcitrant form, and this process was more likely controlled through abiotic rather than biochemical reaction. Therefore, our results indicated that the potential ecological functions of *phoD*-harboring microorganisms with regard to P turnover varied with stand age depending on soil P conditions.

Although many previous studies have been reported that seasonal dynamics of soil bacterial community structures (Griffiths et al., 2003; Spohn et al., 2016; Carson and Zeglin, 2018), the information about the seasonal changes of *phoD*-harboring microbial community structures in forest ecosystems is limited. In the present study, *phoD*-harboring microbial community structures significantly differed between dry and rainy seasons in three stand ages along soil profiles, which were influenced by air temperature and precipitation. Local monthly air temperature and precipitation, which exhibited striking fluctuations between dry and rainy seasons, were used as proxies for monthly changes in soil temperature and moisture. Such could explain seasonal shifts in *phoD*-harboring bacterial communities and is consistent with the findings of previous studies investigating the seasonal dynamics of bacterial communities based on climatic variability (Griffiths et al., 2003; Spohn et al., 2016; Carson and Zeglin, 2018). Seasonal changes in microbial populations could reveal the different niches (Fierer et al., 2007; Samad et al., 2017). The *Bradyrhizobium* and *Methylibium* genera dominated in three stand ages; however, their relative abundances did not vary between the dry and rainy seasons. This was in accordance with many previous studies (Tan et al., 2013; Ragot et al., 2015; Wei et al., 2019). The main reason was that litter of *P. massoniana* decomposed slower and similar root excretion was produced by a dominant tree. This may have had slight effects on seasonal changes of these two dominant genera.

### Phosphorus Use Strategies Implications for Soil Phosphorus Management in *Pinus massoniana* Plantations

P dynamics in forest ecosystems are influenced by soil microbes and the annual tree P uptake (Yang and Post, 2011; Rosling et al., 2016). The sum of annual average MBP and estimated annual tree P requirements exceeded the soil available P pool in our present study, which was in agreement with the earlier studies reports that forest is P-limited (Harpole et al., 2011; Kochian, 2012). We also found that soil P availability was comparable to tree P requirement but was inferior to MBP. The result suggested that P dynamics were controlled by soil microbes rather than by the annual tree P uptake. This is because that the resorbed and internally stored P in woody trees potentially decreases their reliance on P acquired from soil (Rennenberg and Herschbach, 2013). Conversely, microbial growth in subtropical regions may be more limited by soil P availability (Chen et al., 2019), and trees P availability might be drove by microbial dynamics. Because P uptake by microbes is the largest annual P flux in soil P budget (Cole et al., 1977). For example, the annual average MBP was approximately 3-fold the estimated tree P requirements (Rosling et al., 2016), as found in our present study. Microbial growth can immobilized most of the mineralized P in their biomass P (Turner et al., 2013), which leads to low P for plants use.

The lower relative abundances of dominant *phoD*-harboring microorganism genera and ALP activity were accompanied with higher soil P availability with an increase in stand age, which was consistent with our hypothesis. The results suggested the *P. massoniana* growing in young planation soils more relied on *phoD*-harboring microorganisms for the mineralization of Po from mineral soil, than that in old planation soils. This behavior could be closely linked to soil P conditions (Wu et al., 2019a). For example, at young planation soils had low soil P availability, which stimulated the expression of *phoD* (Apel et al., 2007), and accelerate ALP synthesis. This would alleviate P limitation on tree growth *via* the hydrolyzation of Po into Pi. As stands develop, litter input and decomposition increase soil P availability (Wu et al., 2019a) and facilitates tree growth. This would inhibit ALP activity and *phoD* expression (Bergkemper et al., 2016; Wei et al., 2019), resulting in enzyme-P accumulation. Similarly, in our study, we observed higher relative abundance of dominant genera in *phoD*-harboring microorganism communities and ALP activity under low soil P availability in the young planation soils when compared with in the old planation soils. Additionally, trees would reinforce symbiotic interactions with fungi (i.e., ectomycorrhizal fungi and arbuscular mycorrhizal fungi) to acquire P (Rosling et al., 2016) with an increase in stand age. Therefore, extending rotation periods could increase soil P availability and tree production, which is a sustainable plantation management.

## Conclusion

The CaCl_2_-P, citrate-P, enzyme-P, HCl-P, and total available P pools varied seasonally. The P pools were lower in young planation soils but higher in old-mature planation soils, most likely owing to greater litter input and decomposition in the latter planation. Lower P availability levels associated with higher OTU abundance ALP activity in young planation soils than that in old-mature planation soils were found. The results suggested that *phoD*-harboring microorganisms may regulate Po turnover by mineralizing Po into Pi, leading to an increased availability of P under P-poor conditions. Trees can modulate their P use strategies in response to soil P availability based on stand age. Based on our observations, we propose that young-grow trees absorb P derived predominantly from *phoD*-harboring microorganisms following mineralization of Po from mineral soil, while old-growth trees acquire P mainly from litter input and decomposition. More studies are required to confirm these findings. Extending rotation periods could facilitate the maintenance of P supply and support long-term productivity in *P. massoniana* plantations.

## Data Availability Statement

The original contributions presented in the study are included in the article/Supplementary Material, further inquiries can be directed to the corresponding author.

## Author Contributions

YL, FP, and JM conceived and designed the experiments. YL, ML, ZY, TL, JQ, SL, FZ, and ZS performed the experiments. YL analyzed the data. YL and FP wrote the paper. All authors contributed to the article and approved the submitted version.

### Conflict of Interest

The authors declare that the research was conducted in the absence of any commercial or financial relationships that could be construed as a potential conflict of interest.

## Footnotes

^1^http://fungene.cme.msu.edu/FunGenePipeline/

## Supplementary Material

The Supplementary Material for this article can be found online at:

https://www.frontiersin.org/articless/10.3389/fmicb.2020.571209/full#supplementary-material
