# Supplementary material for: Alkaline Phosphomonoesterase-Harboring Microorganisms Mediate Soil Phosphorus Transformation With Stand Age in Chinese Pinus massoniana Plantations
Source: Front Microbiol. 2020 Nov 27;11:571209. doi: 10.3389/fmicb.2020.571209 (PMC7728850; doi:10.3389/fmicb.2020.571209)
Supplement: Supplementary file 1 [file Data_Sheet_1.pdf]

Alkaline phosphomonoesterase-harboring microorganisms mediate soil phosphorus transformation with stand age in Chinese *Pinus massoniana* plantations

Yueming Liang, Mingjin Li, Fujing Pan, Jiangming Ma, Zhangqi Yang, Tianwang Ling, Jiashuang Qin, Shaohao Lu, Fengyue Zhong, Zunrong Song

Supplementary information

Table S1 Stand characteristics in *Pinus massoniana* plantations

Stand type	Mean height (m)	Basal area(m^2 hm^{-2})	Litter biomass (mg g^{-1})	P in roots (mg g^{-1})
YP	5.56	9.3	227.96	0.66
MP	10.79	32.85	254.58	1.98
OP	26.55	55.1	328.33	1.87

Note: YP, 6-year-old, young plantations; MP, 19-year-old, middle-aged plantations; OP, 58-year-old, over-mature plantations.

Table S2 Soil basic physiochemical properties in *Pinus massoniana* plantations

Stand age/Season	Depth	pH	SOC(g kg^{-1})	TN(g kg^{-1})	Mg_{ex}(g kg^{-1})	Ca_{ex}(g kg^{-1})
Dry(0-10 cm)	YP	3.94±0.09Aa	25.58±1.94Aa	2.71±0.97Aa	0.02±0.006Aa	0.84±0.10Aa
	MP	3.96±0.08Aa	32.79±3.57Aa	2.23±0.25Aa	0.02±0.007Aa	1.03±0.17Aa
	OP	3.68±0.03Ba	24.87±2.53Aa	2.34±0.02Aa	0.01±0.0001Aa	0.57±0.03Ba
Dry(10-20 cm)	YP	3.86±0.06Ab	18.29±1.1Aa	1.32±0.16Aa	0.01±0.001Aa	0.51±0.02Ba
	MP	3.91±0.03Ab	17.17±1.49Aa	1.43±0.05Aa	0.01±0.003Aa	0.66±0.03Aa
	OP	3.84±0.03Ab	13.87±3.51Aa	0.91±0.14Ba	0.01±0.003Aa	0.45±0.03Ba
Dry(20-30 cm)	YP	4.03±0.04Ab	8.63±2.18Aa	0.99±0.17Aa	0.005±0.002Aa	0.47±0.03Aa
	MP	4.01±0.02Ab	12.86±0.04Aa	1.35±0.17Aa	0.01±0.0001Aa	0.36±0.11Aa
	OP	3.85±0.03Bba	7.83±2.09Aa	0.67±0.09Ba	0.003±0.003Aa	0.31±0.01Aa
Rainy(0-10 cm)	YP	4.04±0.11Aa	24.96±1.93Aa	1.74±0.21Aa	0.02±0.006Aa	0.89±0.09Aa
	MP	4.14±0.02Aa	26.89±3.53Aa	1.79±0.21Aa	0.02±0.006Aa	1.18±0.21Aa
	OP	3.82±0.08Ba	26.42±6.71Aa	1.44±0.28Aa	0.01±0.003Aa	0.61±0.01Ba
Rainy(10-20 cm)	YP	4.11±0.07Aa	17.31±1.56Aa	1.40±0.27Aa	0.01±0.0001Aa	0.55±0.03Ba
	MP	4.26±0.05Aa	16.80±2.96Aa	1.20±0.14Aa	0.01±0.004Aa	0.67±0.03Aa
	OP	4.02±0.02Ba	14.38±4.77Aa	0.90±0.23Aa	0.01±0.002Aa	0.48±0.03Ba
Rainy(20-30 cm)	YP	4.21±0.03Aa	10.20±0.92Aa	1.03±0.25Aa	0.003±0.003Aa	0.48±0.03Aa
	MP	4.28±0.04ABb	12.87±2.18Aa	0.96±0.08Aa	0.01±0.0001Aa	0.39±0.11Aa
	OP	4.1±0.02Bb	11.96±3.24Aa	0.82±0.18Aa	0.005±0.003Aa	0.34±0.01Aa

Note: Abbreviations see Table S1. SOC, soil organic carbon; TN, total nitrogen; Mg_{ex}, exchangeable Mg; Ca_{ex}, exchangeable Ca. Data (means ± SE, n = 3) followed by different capital case letters are significantly different among stand ages according to Duncan test (p<0.05). Data followed by different lower case letters are significantly different between seasons according to Duncan test (p < 0.05).

Table S3 Two-way ANOVA testing for the effects of age, sampling season and their interactions on soil P fractions of *Pinus massoniana* plantations

Depth (cm)	Factor	$CaCl_2$-P		Citrate-P		Enzyme-P		HCl-P		Total availability P		Microbial biomass P	
		F	*Sig.*	F	*Sig.*	F	*Sig.*	F	*Sig.*	F	*Sig.*	F	*Sig.*
0-10	Age (A)	78.69	**<0.001**	21.33	**<0.001**	19.99	**0.001**	14.41	**<0.001**	3.97	**0.047**	1.09	0.368
	Season (S)	26.85	**<0.001**	19.34	**0.001**	30.72	**<0.001**	0.011	0.916	21.40	**0.001**	62.75	**<0.001**
	A ×S	13.87	**0.001**	8.86	**<0.001**	4.26	**0.04**	22	**<0.001**	0.30	0.745	0.54	0.596
10-20	Age (A)	43.90	**<0.001**	13.98	**<0.001**	16.07	**<0.001**	9.49	**0.002**	5.04	**0.026**	0.35	0.713
	Season (S)	28.84	**<0.001**	20.53	**<0.001**	37.39	**<0.001**	4.36	0.056	0.52	0.486	9.66	**0.009**
	A ×S	9.25	**0.004**	8.17	**<0.001**	3.43	0.067	22.16	**<0.001**	1.64	0.235	0.21	0.810
20-30	Age (A)	37.13	**<0.001**	11.99	**0.001**	8.84	**0.004**	16.87	**<0.001**	1.06	0.376	13.08	**<0.001**
	Season (S)	0.40	0.54	0.39	0.540	12.69	**0.004**	3.44	0.085	6.98	**0.021**	20.82	**<0.001**
	A ×S	1.18	0.34	1.37	0.290	1.40	0.283	16.98	**<0.001**	0.61	0.558	2.23	0.150

Note: F represents the F value of the corresponding factor. Significant effects (*Sig.*) < 0.05 are given in bold.

Table S4 diversity index of *phoD* gene with stand age in *Pinus massoniana* plantations

Season/ Depth	Stand age	Observed OTUs	Chao1	Shannon diversity	Simpson diversity
Dry(0-10 cm)	YP	126±8Aa	149±2Aa	3.16±0.22Ba	0.78±0.02Aa
	MP	123±9Aa	145±4Aa	2.75±0.05Bb	0.76±0.06Aa
	OP	121±3Aa	140±4Aa	3.66±0.26Aa	0.85±0.02Aa
Dry(10-20 cm)	YP	121±2Aa	151±2Aa	3.54±0.14Aa	0.82±0.01Aa
	MP	125±6Aa	154±3Aa	2.73±0.15Ba	0.76±0.02Ba
	OP	115±3Aa	146±2Aa	3.67±0.15Aa	0.86±0.01Aa
Dry(20-30 cm)	YP	116±2Aa	142±2Ab	3.03±0.12Aa	0.75±0.01ABa
	MP	112±4Aa	138±3Aa	2.28±0.07Ba	0.67±0.17Ba
	OP	108±6Aa	137±2Aa	3.32±0.24Aa	0.82±0.09Aa
Rainy(0-10 cm)	YP	128±6Aa	155±7Aa	3.04±0.32Ba	0.77±0.04Aa
	MP	115±5Aa	138±8Aa	3.12±0.10Ba	0.74±0.07Aa
	OP	120±12Aa	138±17Aa	4.14±0.19Aa	0.85±0.06Aa
Rainy(10-20 cm)	YP	117±6Aa	140±7Aa	3.31±0.08Aa	0.79±0.007Aa
	MP	123±5Aa	149±4Aa	2.66±0.07Ba	0.75±0.02Aa
	OP	107±3Aa	138±3Aa	3.09±0.22Aa	0.75±0.04Aa
Rainy(20-30 cm)	YP	127±7Aa	153±2Aa	3.43±0.30Aa	0.81±0.03Aa
	MP	107±3Ba	132±5Ba	2.60±0.26Ba	0.71±0.04Aa
	OP	117±5Aa	140±5Aa	3.57±0.10Aa	0.84±0.02Aa

Note: Abbreviations see Table S1. Data (means ±SE, n = 3) followed by different capital case letters are significantly different among stand ages according to Duncan test (p<0.05). Data followed by different lower case letters are significantly different between seasons according to Duncan test (p < 0.05).

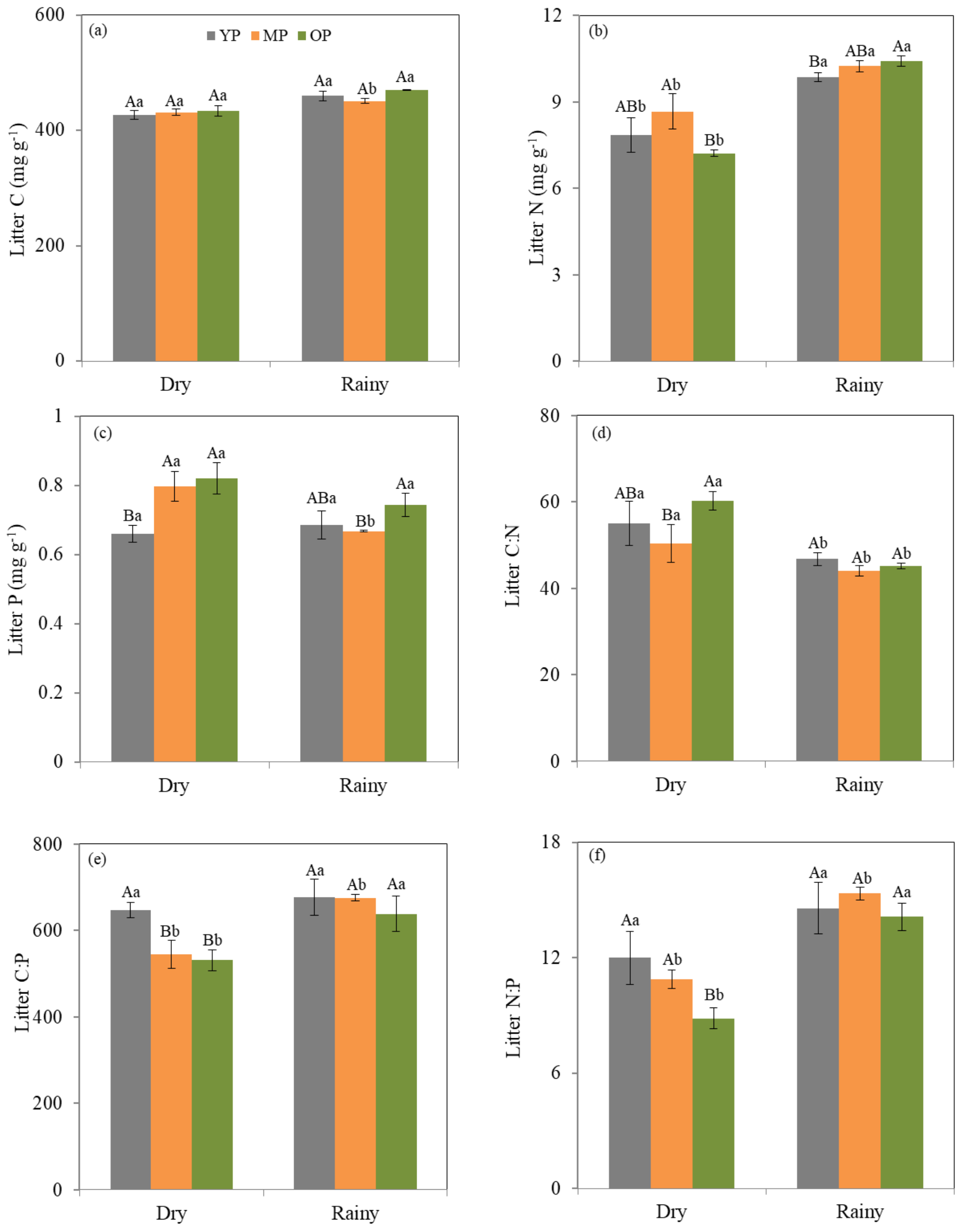

FIGURE S1 Seasonal dynamic of litter C (a), litter N (b), litter P (c), litter C:N (d), litter C:P (e), litter N:P (f) with stand age in *Pinus massoniana* plantations. YP, 6-year-old, young plantations; MP, 19-year-old, middle-aged plantations; OP, 58-year-old, over-mature plantations. Different capital case letters are significantly different among stand ages according to Duncan test ($p < 0.05$). Different lower case letters are significantly different between seasons according to Duncan test ($p < 0.05$).

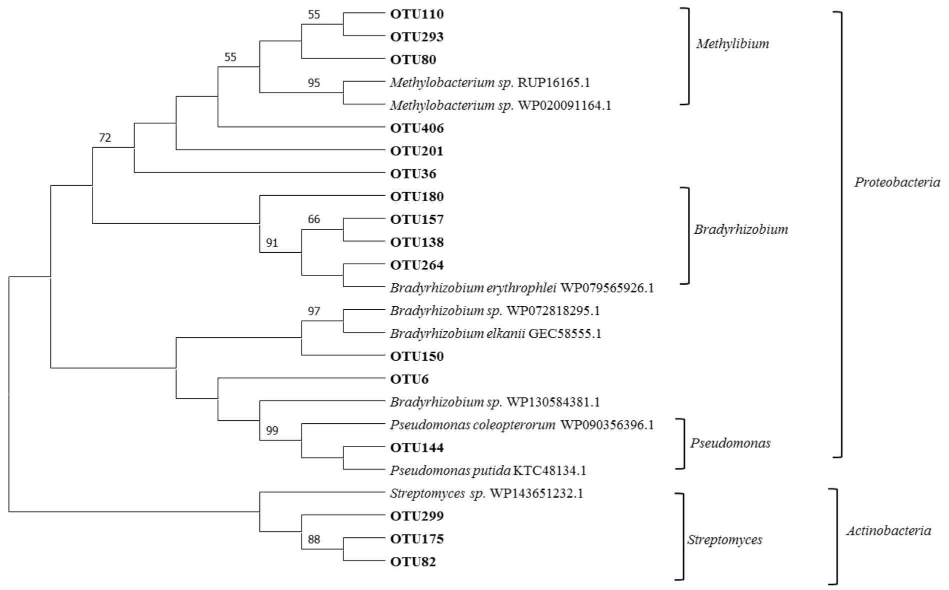

FIGURE S2 Neighbour-joining phylogenetic tree that represents the phylogenetic relationships of the 16 OTUs (relative abundance > 8%) of *phoD* gene to the most closely related identified sequences obtained from the NCBI based on the maximum likelihood method. A bootstrap analysis was performed by 1000 trials. The bar indicates an estimated sequence divergence.

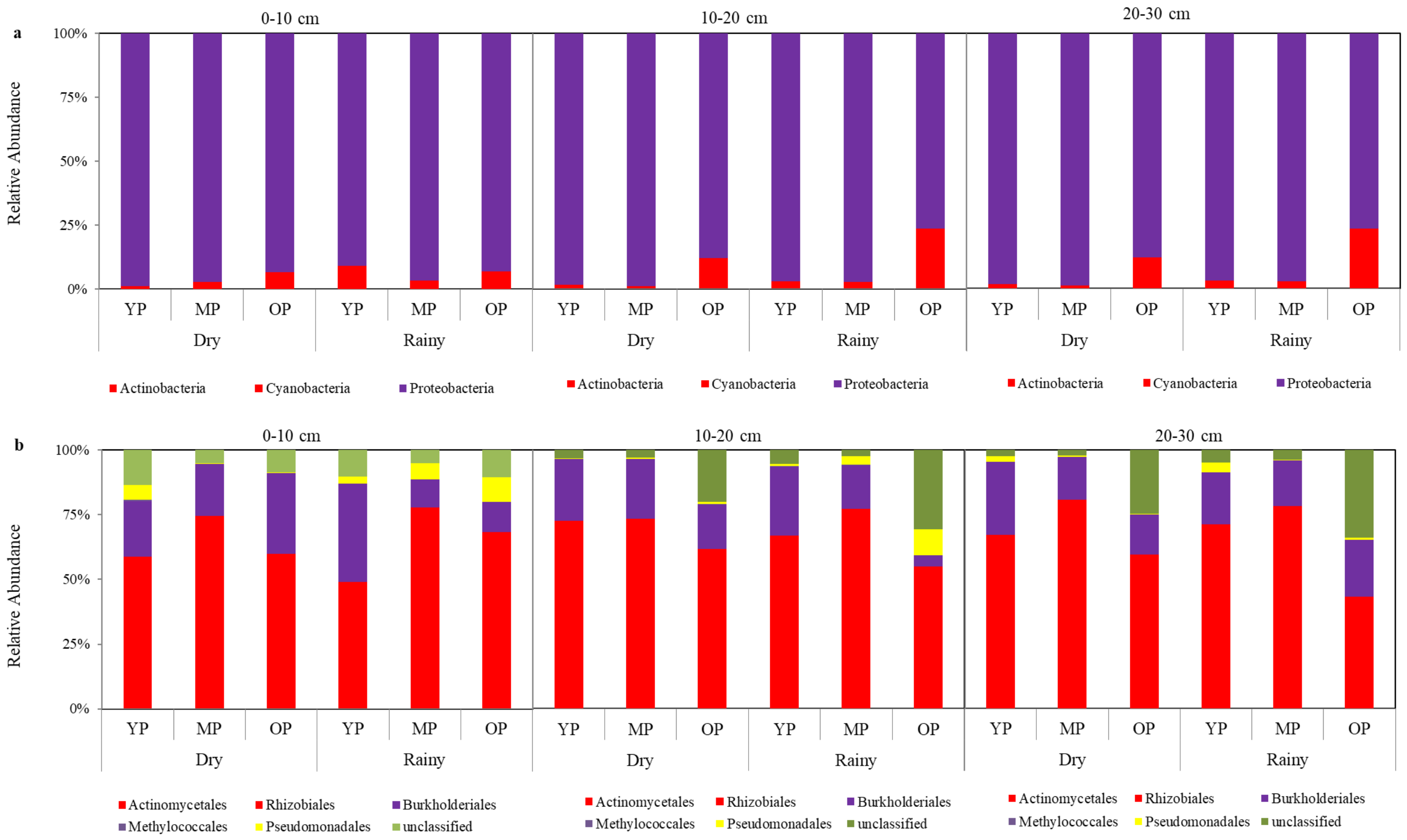

FIGURE S3 Taxonomic composition of *phoD*-harboring microorganism communities at the phylum (a) and order (b) level at different soil layer and stand age of *Pinus massoniana* plantations. YP, 6-year-old, young plantations; MP, 19-year-old, middle-aged plantations; OP, 58-year-old, over-mature plantations.

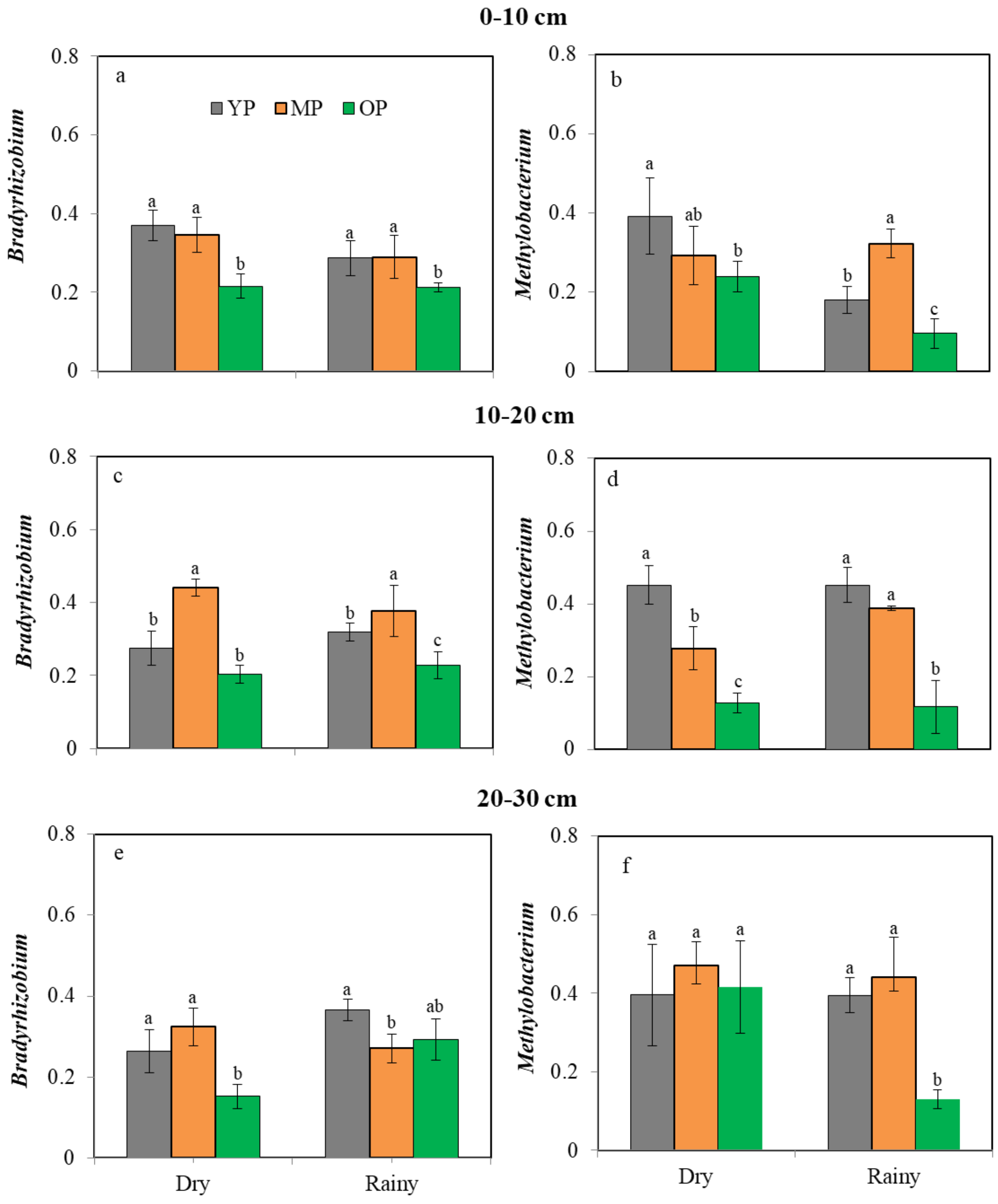

FIGURE S4 Relative abundance of the two most abundant genera (> 8%) in different stand ages and seasons of *Pinus massoniana* plantations. Different case letters are significantly different among stand ages according to Duncan test ($p < 0.05$). YP, 6-year-old, young plantations; MP, 19-year-old, middle-aged plantations; OP, 58-year-old, over-mature plantations.

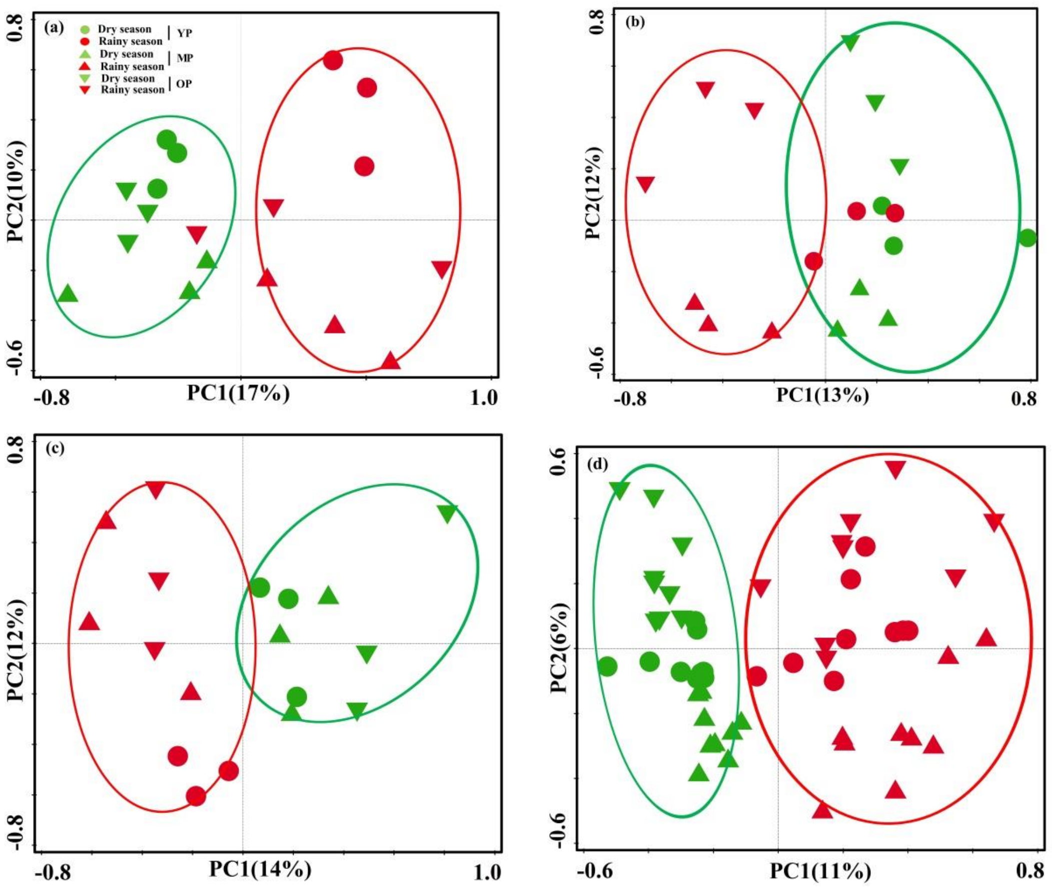

FIGURE S5 Principal component analysis (PCA) analysis of seasonal dynamic of *phoD*-harboring microorganism communities at 0-10 cm (a), 10-20 cm (b), 20-30 cm (c) and 0-30 cm (d). YP, 6-year-old, young plantations; MP, 19-year-old, middle-aged plantations; OP, 58-year-old, over-mature plantations.
